# Targeting Src family kinase member Fyn by Saracatinib attenuated liver fibrosis in vitro and in vivo

**DOI:** 10.1038/s41419-020-2229-2

**Published:** 2020-02-12

**Authors:** Guifang Du, Jing Wang, Ting Zhang, Qiang Ding, Xiaodong Jia, Xueke Zhao, Jinke Dong, Xinrui Yang, Shanshan Lu, Cuihong Zhang, Ze Liu, Zhen Zeng, Rifaat Safadi, Ruizhao Qi, Xin Zhao, Zhixian Hong, Yinying Lu

**Affiliations:** 1 Comprehensive Liver Cancer Centre, the Fifth Medical Center of PLA General Hospital, Beijing, China; 2 State Key Laboratory of Toxicology and Medical Countermeasures, Beijing Institute of Pharmacology and Toxicology, Beijing, China; 3 Department of Medicine, University of Alabama at Birmingham, Birmingham, AL USA; 4 Guizhou Medical University, Guizhou, China; 5 Hadassah Medical Organization, Hadassah Hebrew University Medical Center, Jerusalem, Israel; 6 Department of Hepatobiliary surgery, the Fifth Medical Center of PLA General Hospital, Beijing, China; 7 Center for Synthetic and Systems Biology, Tsinghua University, Beijing, China

**Keywords:** Experimental models of disease, Preclinical research

## Abstract

Recent studies suggest that Src family kinase (SFK) plays important roles in systemic sclerosis and pulmonary fibrosis. However, how SFKs contributed to the pathogenesis of liver fibrosis remains largely unknown. Here, we investigated the role of Fyn, a member of SFK, in hepatic stellate cell (HSC) activation and liver fibrosis, and evaluated the anti-fibrotic effects of Saracatinib, a clinically proven safe Fyn inhibitor. Fyn activation was examined in human normal and fibrotic liver tissues. The roles of Fyn in HSC activation and liver fibrosis were evaluated in HSC cell lines by using Fyn siRNA and in Fyn knockout mice. The effects of Saracatinib on HSC activation and liver fibrosis were determined in primary HSCs and CCl_4_ induced liver fibrosis model. We showed that the Fyn was activated in the liver of human fibrosis patients. TGF-β induced the activation of Fyn in HSC cell lines. Knockdown of Fyn significantly blocked HSC activation, proliferation, and migration. Fyn deficient mice were resistant to CCl_4_ induced liver fibrosis. Saracatinib treatment abolished the activation of Fyn, downregulated the Fyn/FAK/N-WASP signaling in HSCs, and subsequently prevented the activation of HSCs. Saracatinib treatment significantly reduced the severity liver fibrosis induced by CCl_4_ in mice. In conclusions, our findings supported the critical role of Fyn in HSC activation and development of liver fibrosis. Fyn could serve as a promising drug target for liver fibrosis treatment. Fyn inhibitor Saracatinib significantly inhibited HSC activation and attenuated liver fibrosis in mouse model.

## Introduction

Liver fibrosis is a global health problem and a critical process in liver diseases as well as a major risk factor for progression to cirrhosis and hepatocellular carcinoma^1^. The progression and resolution of fibrosis is a complex process involving the interaction between parenchymal and nonparenchymal cells. Chronic hepatocyte death and inflammation caused by viral infection, excessive alcohol intake, and nonalcoholic steatohepatitis are considered as a triggering event in liver fibrosis^2,3^. It is widely recognized that activated hepatic stellate cells (HSCs) play a pivotal role in the development of liver fibrosis. In response to liver damage, HSCs undergo a process of activation that acquires a myofibroblast-like phenotype with increased cell proliferation, the synthesis of excess extracellular matrix (ECM), the secretion of proinflammatory cytokines, and the capacity to migrate and contract^4^. Several cytokines are critical in HSC activation, one of the most important cytokine is TGF-β^3^. TGF-β induces the activation of the downstream molecules such as SMAD, ERK, AKT signaling and promotes the transition of quiescent phenotype to myofibroblast-like phenotype in HSCs^5^. So, targeting TGF-β signaling is a very effective strategy for therapy of liver fibrosis^6^.

Src kinase family, is a family of nonreceptor tyrosine kinases that plays key roles in regulating signal transduction. The members of Src family kinases (SFKs) including Src, Yes, Fyn, Fgr and so on^7^. Previously, we and others showed that, TGF-β induced the activation of SFKs including Src and Fyn^8–12^. SFKs, especially Fyn was considered essential for the bioactivity of TGF-β, evidenced by the absence of Fyn abolished cortical actin ring depolymerization and migration in mast cells^9^. In addition, blockade of Fyn with siRNA reduced the ability of TGF-β to repress E-cadherin expression in A549 lung cancer cells^10^. SFKs has been reported to play important roles in lung fibrosis, renal fibrosis, and systemic sclerosis^8,12–14^. Blockade of SFKs, such as Src and Fyn, was associated with reduced ECM production in fibroblasts and improved tissue fibrosis^8,12,13^. Taken together, the activation of SFKs by upstream factors such as TGF-β, was critical in the fibrosis of lung and kidney. However, how SFKs contribute to the activation of HSCs and the development of liver fibrosis remains elusive.

Saracatinib (AZD0530), is an orally bioavailable aniline-quinazoline that has been shown to be a potent SFK/Abl dual-kinase inhibitor. Saracatinib inhibited Src activation and prostate cancer cell growth in vitro and in an in vivo murine xenograft model^15^. Although the phase II clinical trial failed to show the benefit against prostate cancer in patients^16^, the phase I clinical trial confirmed the feasibility and tolerability of Saracatinib treatment in patients^17^. Our previous study indicated that Saracatinib blocked TGF-β-induced Src kinase activation in a dose-dependent manner and reduced ECM production in lung fibroblasts. Saracatinib also attenuated the severity of lung fibrosis in the bleomycin murine lung fibrosis model^8^. However, whether Saracatinib can benefit liver fibrosis or not is unknown.

In this study, we investigated the role of Fyn, one of the SFKs which is highly expressed in HSCs and activated in fibrotic livers, in the development of liver fibrosis. We also evaluated the in vitro and in vivo anti-liver fibrotic activities of SFK inhibitor Saracatinib, which was proven safe in clinical trials.

## Materials and methods

### Materials

Saracatinib (CAS: 379231-04-6) was purchased from Selleck Chemicals (USA). TGF-β (CAT. 240-B-010-RND) was purchased from R&D Systems. Male C57BL/6 mice were bought from Beijing Vital River Laboratory Animal Technology Co., Ltd. Fyn knockout C57BL/6 mice (male, 8 weeks) were purchased from Jackson laboratory.

### Patient enrollment

Three normal and three liver fibrosis tissues sample were obtained from the Department of Hepatobiliary Surgery of the Fifth Medical Center of PLA General Hospital (Beijing, China). Informed consent in writing was obtained from patients. This study protocol was approved by the review committee of the Fifth Medical Center of PLA General Hospital (2014046D).

### Histological and immunohistochemical studies

Liver samples were formalin-fixed, paraffin-embedded and sectioned, and processed routinely for H&E, Masson, and Sirius red staining. The antibody for immunohistochemical staining of phospho-Fyn (P-Fyn Y416) was purchased from Cell Signaling Technology (#6943).

### Cell culture

Immortalized human (LX-2) and rat (HSC-T6) HSC lines were used according to the previous reports^18,19^. LX-2 cell line was kindly provided by Professor Liying Li. HSC-T6 cell was purchased from Bio Tech Co., Ltd (#CL-0116). Mouse primary HSC was isolated according to the procedures described previously^20^. Cells and mouse primary HSC in DMEM (Hyclone, USA), and all were supplemented with 10% (vol/vol) FBS, 100 units/mL penicillin G, 100 μg/mL streptomycin sulfate, and 2 mM l-glutamine. All cells were cultured in a 37 °C humidified incubator under an atmosphere of 5% (vol/vol) CO_2_ in air.

### TGF-β treatment

For TGF-β induction, HSCs were serum deprived for 4 h, and then treated with Saracatinib for 4 h, and followed by stimulation with recombinant human TGF-β 10 ng/mL for 20 min.

### Co-immunoprecipitation

For co-immunprecpititation experiments, whole-cell extracts were prepared in lysis buffer (Cell Signaling) with protease inhibitor complex (Roche diagnostics) and subsequently immunoprecipitated by anti-Src Family (phosphor Y416) antibody (CST, #6943 S) which immobilized on protein A/G PLUS-Agarose (Santa Cruz Biotechnology). The immunoprecipitates were resolved by a 10% SDS-PAGE and immunoblotted with antibodies listed below anti-Fyn (CST, #4023 T), anti-Src (CST, #2123 T), anti-Lyn (CST, #2796 T), anti-Lck (Santa Cruz Biotechnology), and anti-Hck (CST, #14643 S) antibodies, respectively.

### Western blotting

For western blot, after denaturation, the samples were loaded into narrow wells of 10% SDS-PAGE, and then the gel was run to separate proteins. Proteins were wet transferred to a PVDF membrane, and the membrane was blocked by incubation with 5% skim milk at room temperature for 2 h. Then the membrane was incubated with primary antibodies at 4 °C overnight. After pouring off first antibodies, the membrane was rinsed briefly with TBST buffer three times, 15 min for per time and then secondary antibody was added at appropriate dilution. After rocking gently for 2 h, the membrane was again washed as before. Antibodies used for western blot testing include: anti-FAK (Thermo, #PA5-17591), anti-p-FAK(397) (Invitrogen, #700255), anti-N-WASP (R&D, #AF3854), anti-p-N-WASP (256) (abcam, #ab23395), anti-α-SMA (abcam, #ab5694), anti-collagen I (abcam, #ab138492), anti-p-Fyn(530) (GeneTax, #GTX3215), anti-ERK1/2 (Santa cruz, #SC-514302), anti-p-ERK 1/2 (Santa cruz, #SC-136521), anti-p-Akt (Santa cruz, #SC-271966), and anti-Akt (Santa cruz, #SC-5298).

### Isolation and culture of primary mouse HSCs

Primary mouse hepatocytes were isolated from mouse liver according to a previous protocol^21^. In brief, the livers of the mice were first perfused in situ via the portal vein with Hank’s balanced salt solution (HBSS) supplemented with 0.5 mM EGTA and 25 mM HEPES at 37 °C. Then, the buffer was replaced with 0.1% collagenase solution in HBSS (containing 4 mM CaCl_2_ and 0.8 mM MgSO_4_). After a few minutes of perfusion, the liver was excised rapidly from the body cavity and dispersed into cold HBSS. The cell suspension generated was filtered through a sterile 70-mm pore size nylon cell strainer (Falcon; BD Biosciences) and spun three times at 30 g for 4 min. The pellets were suspended in RPMI 1640 medium containing 10% fetal bovine serum plus 10% horse serum for primary hepatocyte culture.

### Liver fibrosis induced by CCl_4_

Male C57BL/6 mice, aged 6-8 weeks, were purchased with the approval of the Institutional Ethics Committee. Each experimental group contained three mice. This animal study had obtained the approval of committee of the Fifth Medical Center of PLA General Hospital﻿. For CCl_4_-induced liver fibrosis study, all fibrotic experimental groups received 40% CCl_4_ with in i.p. injection (2 ml/kg/body weight dissolved in 100 μl of olive oil) three times per week. Liver fibrosis was induced by CCl_4_ administration for 10 weeks. The treatment group was administered with CCl_4_ for 4 weeks and then treated with Saracatinib (15 or 40 mg/kg) daily, together with CCl_4_ injection for another 6 weeks, whereas controlled mice only received olive oil for 10 weeks.

### Effects of Saracatinib on cell proliferation

Cell viability was evaluated using a nonradioactive cell counting kit (CCK-8, Dojindo, Kamimashiki-gun, Kumamoto, Japan) according to the manufacturer’s instructions. Primary mouse HSCs, and HSCT6, and LX-2 were seeded in a 96-well plate at a density of 3000 cells per well, and after every 24 h, the CCK-8 reagent was added to each well and the plates were incubated for an additional 1 h at 37 °C. Cell viability was measured as the absorbance at 450 nm with an Elx800™ spectrophotometer (BioTek, Winooski, VT, USA).

### HSC migration assay

The migratory properties of HSC cells were assessed by wound-healing assay. Cells were seeded at a density of 8 × 10^5^ cells/well into a six-well plate and allowed to reach confluence. The layer of cells was then scraped with a fine gauge needle to create a wound of ~1500 μm. Images of the wound were recorded under a phase contrast microscope at different times (0, 6, 12, and 24 h). Wound closure/cell migration was evaluated with Image J software.

### RNA interference

Transfections were performed on cells with a Fyn siRNA concentration of 100 nM. SiRNA were transfected with lipofectamine 2000 (Invitrogen, Carlsbad, CA) according to the manufacturer’s recommendations. The sequence of siRNA are described in Supplementary Table 1.

### Statistical analysis

Continuous variables are presented as mean ± standard deviation (SD) and categorical data are presented as number (percentage) as appropriate. To compare values between two groups, the Student’s *t* test was used. All tests were two-sided and a *p* value < 0.05 was considered significant. Most of the experiments were repeated in three independent trials with similar results, and representative images are included in this article.

## Results

### Fyn was abnormally activated in clinic fibrotic liver tissues

**Fig. 1:**
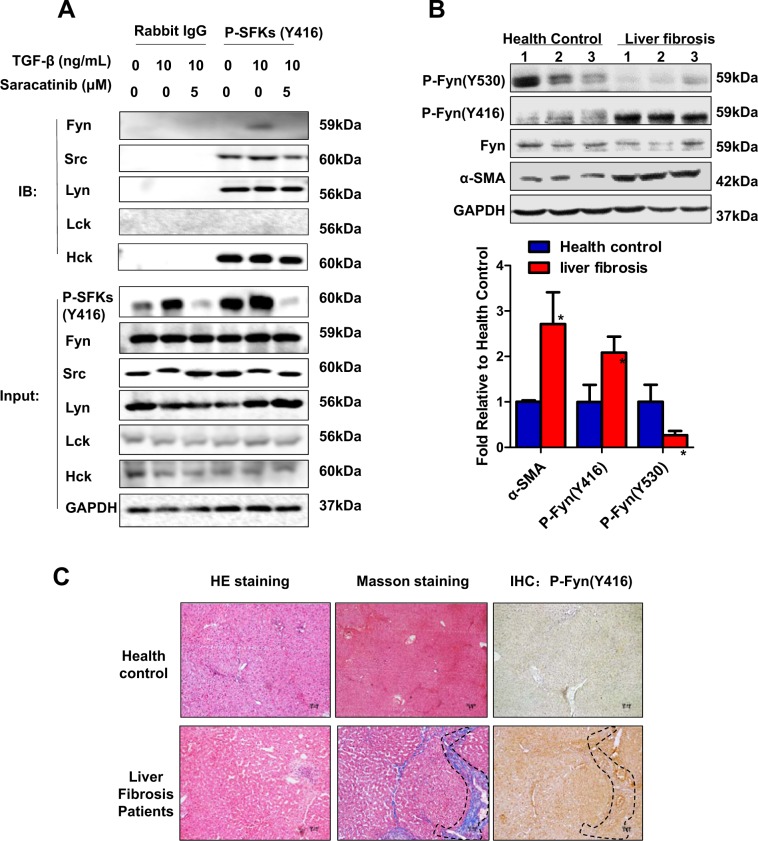
Fyn was abnormally activated in fibrotic liver tissues. **a** Co-immunoprecipitation of phosphorylation Src (Y-416) family, subsequently western blotting analysis of each member of Src family (FYN, SRC, LYN, LCK, HCK). **b** Western blotting analysis of Fyn expression and phosphorylation level of Fyn (Y-416) from normal and liver fibrosis biopsies. (Mean ± SD; *N* = 3, *p < 0.05 by Student’s *t* test). **c** Representative sections of H&E staining, Masson staining, and IHC on p-Fyn (Y416) from paraffin-embedded sections of healthy, fibrotic, and cirrhotic liver tissues. Dash line indicated fibrotic area.

To determine the role of SFKs in the development of liver fibrosis, we treated human HSC cell line LX-2 cells with profibrogenic cytokine TGF-β (10 ng/ml). TGF-β greatly increased the levels of pY416 SFKs detected by an antibody (P-SFKs Y416) that can recognize the phosphorylation site of Y416 in most SFKs including Fyn, Src, Lyn, Lck, and Hck (Fig. 1a). We then used P-SFKs Y416 antibody in a co-IP assay to clarify which member(s) of SFKs was activated by TGF-β in LX-2 cells, as shown in Fig. 1a, only Fyn but no other members of SFKs were significantly increased with TGF-β treatment, which suggested Fyn was responsible for TGF-β-induced SFKs activation in LX-2 cells. Actually, the expression of Fyn at transcriptional level was the highest in the src family, compared with other members (Fig. S1). Next, we tested whether Fyn was activated in human liver fibrosis patients. As shown in Fig. 1b, we observed a significantly increase of pY416-Fyn (activated site) in fibrotic liver compared with health control, while the total Fyn levels were comparable in the two groups. On the contrary, pY530-Fyn (inhibitory site) were decreased. As expected, α-SMA, the activation marker of HSCs was highly expressed in fibrotic liver. To clarify the cell source of pY416-Fyn, we performed serial section staining for H&E, Masson staining, and immunohistochemical staining for pY416-Fyn. As shown in Fig. 1c, the fibrotic areas showed highest pY416-Fyn staining, which suggested that activated HSCs are the main source of activated Fyn in the liver with fibrosis.

### Fyn signaling contributed to HSC activation

**Fig. 2:**
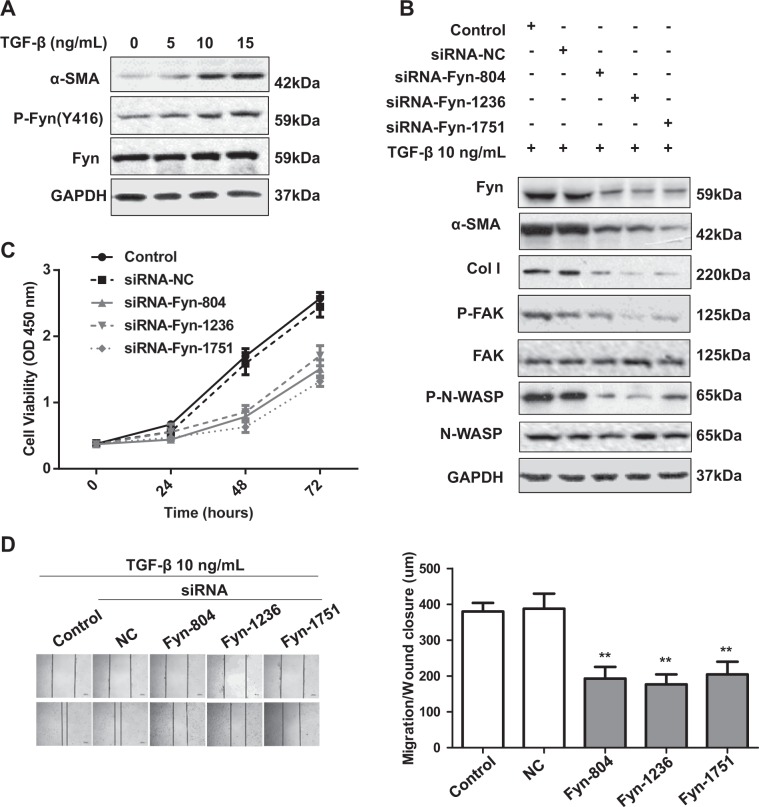
Fyn signaling contributed to HSC activation. **a** The pY416-Fyn and α-SMA levels of LX2 were checked upon TGF-β treatment. **b** LX-2 cells were transfected with si-Fyn or control siRNA for 48 h then treated with TGF-β (10 ng/mL) for the indicated time intervals. The cell extracts were then subjected to western blotting analyses. **c** The effect of knockdown Fyn with siRNA on LX-2 cells proliferation. **d** The effect of knockdown Fyn with siRNA on LX-2 cells migration. Data are from a representative experiment that was repeated three times with similar results. (Mean ± SD, **p < 0.01 by Student’s *t* test).

We next asked whether the activation of Fyn was required for HSC activation and ECM production. The human HSC cell line LX-2 cells were treated with TGF-β (10 ng/ml). As expected, the activation marker for HSCs, α-SMA, was greatly increased with TGF-β treatment, meanwhile, the pY416-Fyn levels were also increased upon TGF-β treatment (Fig. 2a). To clarify the role of Fyn in HSC activation, we effectively knocked down Fyn by siRNA, as shown in Fig. 2b, knockdown Fyn suppressed the increase of α-SMA, collagen I, and phosphorylated FAK, and N-WASP in LX-2 cells induced by TGF-β. Moreover, enhanced proliferation and migration ability of LX-2 by TGF-β treatment was blocked by Fyn siRNA (Fig. 2c, d).

**Fig. 3:**
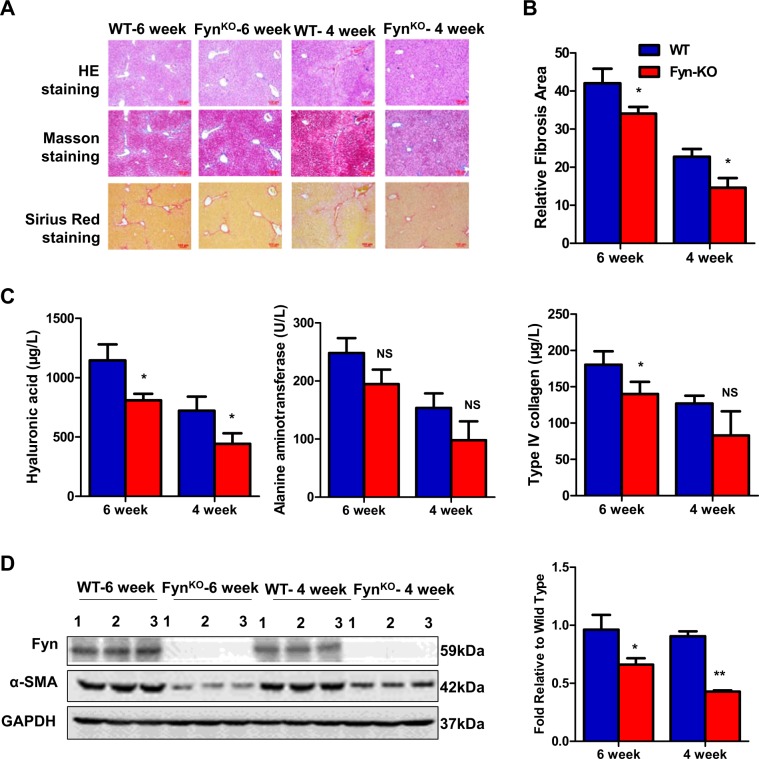
Fyn deficiency in vivo attenuated liver fibrosis induced by CCl_4_. In vivo test was performed in 6-week-old C57BL/6 mice by CCl_4_ intraperitoneal injection for 4 or 6 weeks. **a** Representative photograph of microphotograph of H&E staining, Masson staining, and Sirius red staining paraffin-embedded sections of liver tissues (scale bars, 100 μm). **b** Statistic analysis on Sirius red staining, relative area of liver fibrosis was calculated with Image-Pro Plus on three different fields of view. **c** Serum chemistries analysis on the level of hyaluronic acid, alanine aminotransferase, and type IV collagen. **d** Western blot analysis showed that of Fyn and a-SMA in Fyn WT and KO mouse liver. (Mean ± SD; *N* = 3, *p < 0.05, **p < 0.01, by Student’s *t* test).

We then further evaluated the role of Fyn HSC activation in an in vivo mouse model of liver fibrosis. WT and Fyn KO mice received chronic CCl_4_ injections for 4 and 6 weeks. WT mice developed significant liver fibrosis after chronic CCl_4_ treatment evidenced by H&E, Masson staining, and Sirius red staining as shown in Fig. 3a, b. In contrast, Fyn KO mice showed much less collagen staining compared with WT mice (Fig. 3a, b). In consistent with these staining results, other markers of liver fibrosis, such as liver hyaluronic acid levels, alanine aminotransferase, type IV collagen content, and liver α-SMA levels were all lower in Fyn KO mice compared with WT mice (Fig. 3c, d). All these data supported the critical role of Fyn in HSCs activation and development of liver fibrosis.

### Saracatinib suppressed HSC activation in vitro

**Fig. 4:**
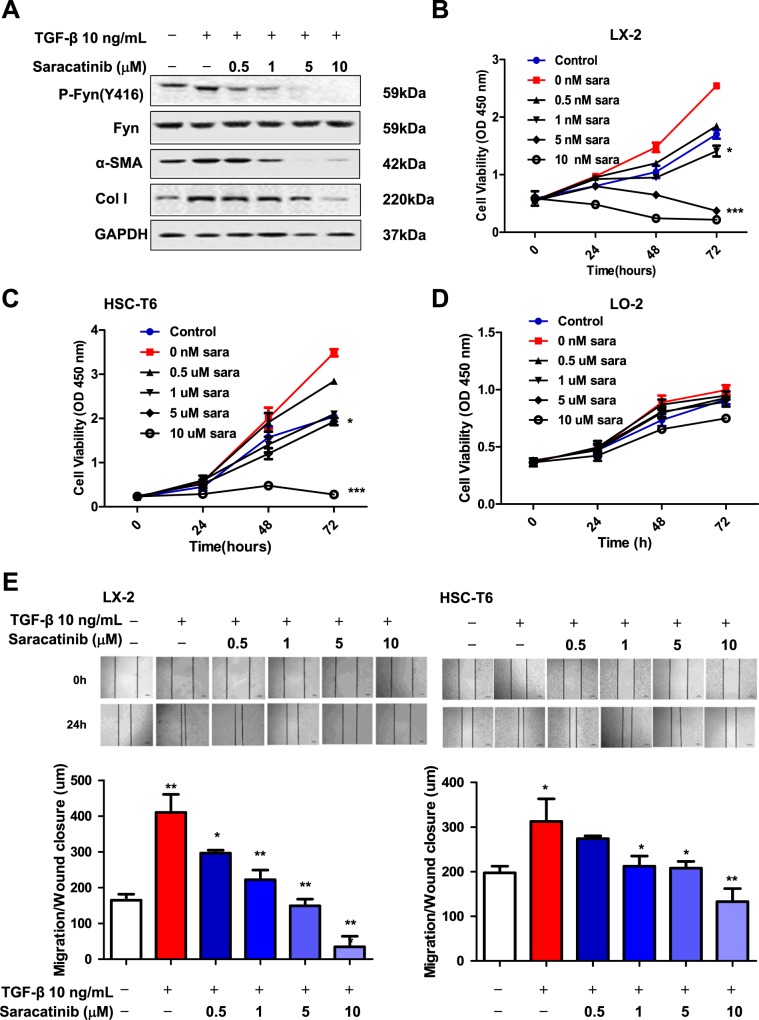
The effect of Saracatinib on activation, growth and migration in HSC cell lines. **a** LX-2 cells were treatment with Saracatinib for 24 h, and stimulated by TGF-β, the expression of HSC activation marker α-SMA, collagen I, and the P-Fyn (416) were detected by western blot, α-SMA and collagen was significantly decreased. Effect of Saracatinib on HSC-T6 (**b**), LX-2 (**c**), and LO-2 (**d**) cells. The cells were cultured for 72 h in the presence of various concentrations of Saracatinib. Proliferation was measured by the CCK-8 kit assay. Results were representative of three experiments and each concentration was repeated six times in each experiment. **e** Saracatinib inhibited the migration of activated HSC determined by the wound-healing assay. HSC-T6 and LX-2 were incubated in the presence of various concentration of Saracatinib for 24 h. Values are expressed as a migrating area of untreated control from three independent experiments. (Mean ± SD; *N* = 3, *p < 0.05, **p < 0.01, ***p < 0.001 by Student’s *t* test).

As our data indicated that Fyn can be considered as an attractive drug target for the liver fibrosis treatment, we evaluated the possible therapeutic effects on liver fibrosis of a clinically proven safe small molecule Fyn inhibitor Saracatinib. Our data also supported the safety of Saracatinib in mice (Fig. S2). Saracatinib significantly reduced the elevated levels of Fyn by TGF-β treatment in LX-2 cells captured by P-SFKs Y416 antibody (Fig. 1a). Moreover, we showed that Saracatinib dose dependently blocked the phosphorylation of Y416 of Fyn (Fig. 4a). These data suggested that Saracatinib may directly target Fyn activation. Also, the activation markers of HSCs, such as α-SMA and collagen I, were reduced by Saracatinib treatment in a dose-dependent manner. In addition, the presence of Saracatinib reduced the cell viability in both human HSC cell line LX-2 cells and rat HSC cell line HSC-T6 cells as determined by CCK-8 assay, In contrast, Saracatinib only showed minor effects on the cell viability of human fetal hepatocyte line LO2 cells (Fig. 4b–d). Further studies indicated that the viability inhibition of Saracatinib on HSCs was not by inducing apoptosis (Fig. S3). Next, we checked the effects of Saracatinib on HSC cell migration by using wound-healing assay. As shown in Fig. 4e, the wound closure was promoted by TGF-β and the effects of TGF-β were attenuated by Saracatinib dose dependently in LX-2 cells and HSC-T6 cells. These data supported that the TGF-β-mediated activation and migration of HSCs can be blocked by Saracatinib.

**Fig. 5:**
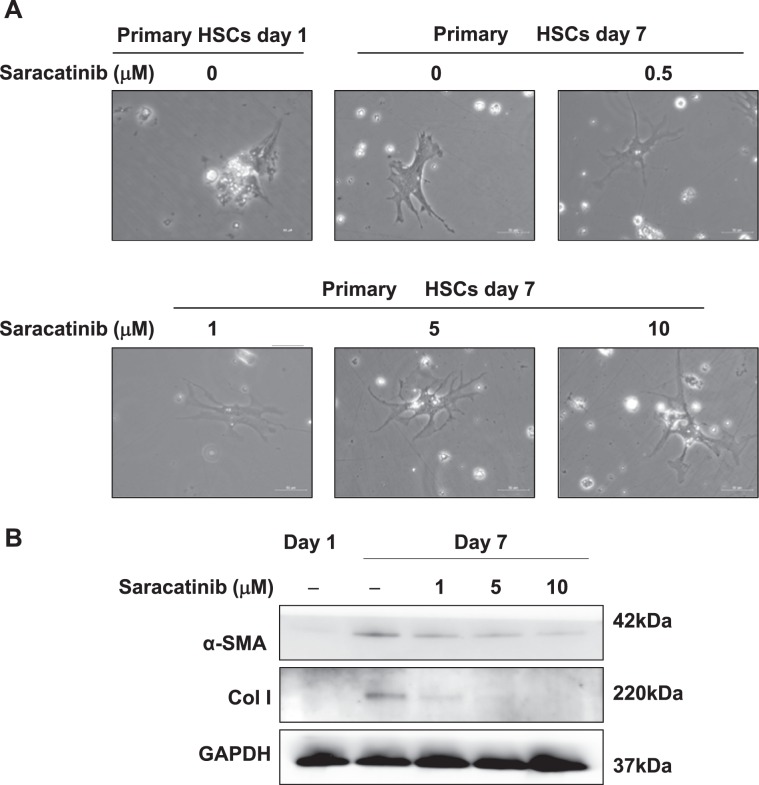
The effect of Saracatinib on primary HSC morphology and activation. **a** The representative photos of morphology change on primary mouse HSCs on day 7 with or without Saracatinib treatment. **b** Western blot checked the expression of α-SMA and collagen I on primary HSCs with or without Saracatinib treatment.

We then tested the potential anti-fibrotic effects of Saracatinib in freshly isolated mouse HSCs. HSCs isolated from mice were cultured for 7 days, in control group, the HSCs showed a typical myofibroblast morphology and high levels of HSC activation markers α-SMA and collagen I expression (Fig. 5a, b). The presence of Saracatinib preserved the features of quiescent HSCs, such as lipid vesicles at perinuclear sites and astral-like morphology (Fig. 5a). The upregulation of α-SMA and collagen I was also reduced with Saracatinib treatment (Fig. 5b). Taken together, Saracatinib blocked the activation of Fyn and prevented the activation of both HSC cell lines and freshly isolated HSCs in vitro.

### Saracatinib suppressed Fyn/FAK/N-WASP pathway

**Fig. 6:**
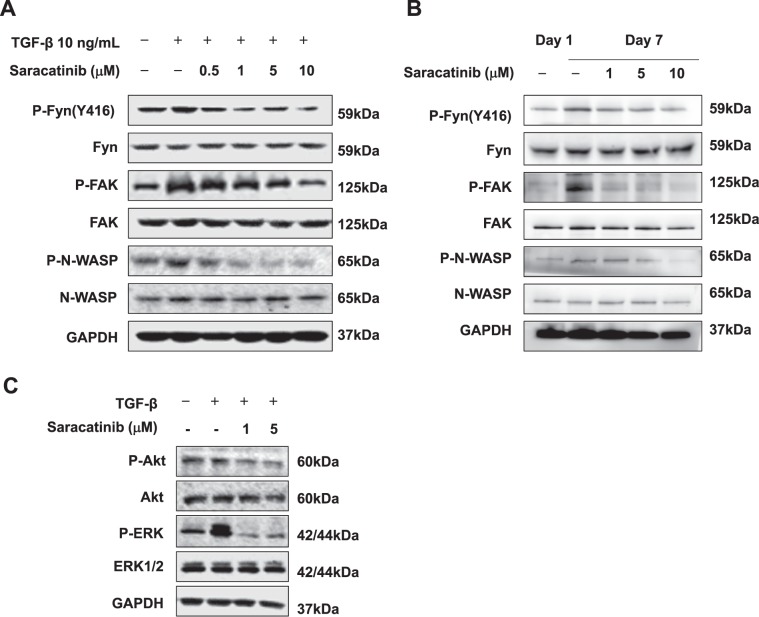
Saracatinib suppressed Fyn/FAK/N-WASP pathway. Western blot analysis on the expression of phosphorylated Fyn, FAK, and N-WASP in LX-2 cells (**a**) and mouse primary HSCs (**b**) with Saracatinib, following treatment with 10 ng/mL TGF-β. **c** Western blot analysis showed that Saracatinib suppressed the expression of phosphorylated Akt and ERK in LX-2 cells treated with 10 ng/mL TGF-β. Data are from a representative experiment that was repeated three times.

Cross-talk between Src kinase and FAK is necessary to sustain FAK activity^8^, which was necessary for the phosphorylation of N-WASP and formation of α-SMA filaments^22^. We tested whether the similar signaling pathway was involved in Fyn mediated HSC activation. We showed that the phosphorylation of both FAK and N-WASP was blocked when Fyn activation was inhibited by Saracatinib (Fig. 6a). Similar results were also observed in mouse primary HSCs (Fig. 6b). Furthermore, Saracatinib reduced the activation of downstream signaling of FAK, such as ERK and AKT signaling, which were critical in promoting cell proliferation and survival (Fig. 6c).

### Saracatinib alleviates murine liver fibrosis induced by CCl_4_

**Fig. 7:**
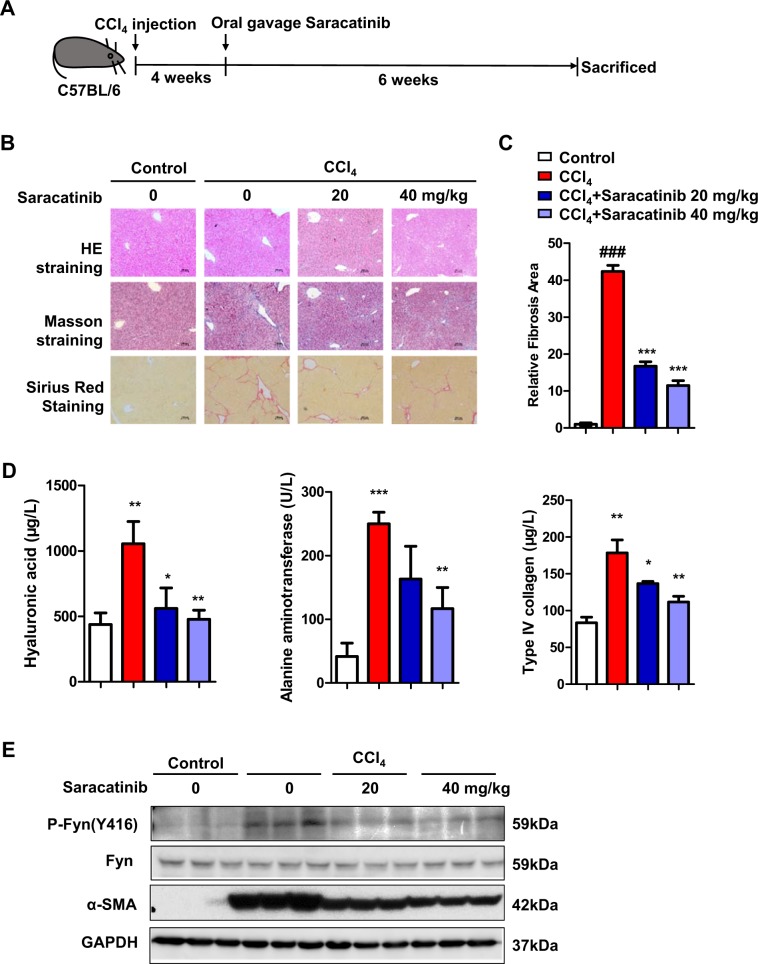
Saracatinib alleviates murine liver fibrosis induced by CCl_4_. **a** Schematic diagram of animal study. The liver fibrosis model was performed in 6-week-old C57BL/6 mice by CCl_4_ intraperitoneal injection for 4 weeks. Then Saracatinib was administrated by gavage once a day for another 6 weeks mice were randomly divided into four groups (*N* = 6–10 in every group): control, CCl_4_ injection, CCl_4_ injection + Saracatinib 20 mg/kg and CCl_4_ injection + Saracatinib 40 mg/kg. **b** Representative photograph of H&E staining, Masson staining, and Sirius red staining paraffin-embedded sections of liver tissues (scale bars, 100 μm). **c** Statistical analysis on Sirius red staining, relative area of liver fibrosis was calculated with Image-Pro Plus on three different fields of view from 6 to10 mice. **d** Serum chemistries analysis on hyaluronic acid, alanine aminotransferase, and type IV collagen. **e** Western blot analysis showed that of Saracatinib also significantly suppressed the expression of P-Fyn (Y416), and α-SMA in mouse liver. (Mean ± SD; *N* = 3, *p < 0.05, **p < 0.01, and ***p < 0.001, by Student’s *t* test).

The prominent anti-fibrotic effects of Saracatinib in vitro made us further explore its beneficial effects in vivo for liver fibrosis. Saracatinib (20 and 40 mg/kg) was administrated by gavage once a day for 6 weeks after 4 weeks of CCl_4_ injections. As shown in Fig. 7a, b, Saracatinib significantly improved liver fibrosis evidenced by H&E, Masson, and Sirus red staining. The increase of liver hyaluronic acid levels and Type IV collagen content by chronic CCl_4_ treatment was significantly attenuated by Saracatinib (Fig. 7c). Western blot results indicated that the activation of Fyn in chronic CCl_4_ treated mice was inhibited by Saracatinib, the elevated α-SMA was also blocked in mice with Saracatinib gavage (Fig. 7d). These data confirmed Saracatinib can effectively alleviate liver fibrosis in murine model.

## Discussion

**Fig. 8:**
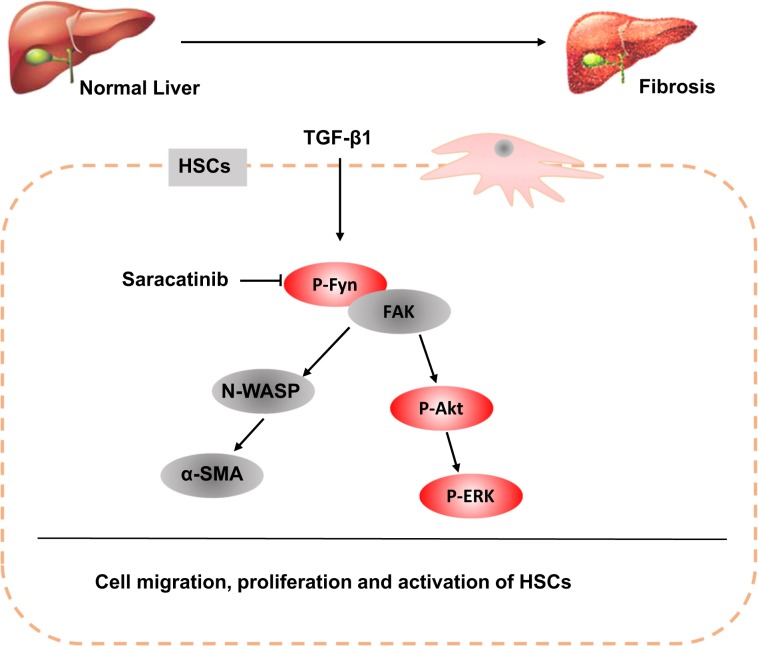
A schematic model of the anti-fibrotic mechanism of Saracatinib.

In this study, we showed the critical role of Fyn in the activation of HSCs and the development of liver fibrosis. As summarized in Fig. 8, Fyn was activated in HSCs with TGF-β stimulation, chronic CCl_4_ treated fibrotic mouse liver, and liver samples from human liver fibrosis patients. Activated Fyn promoted HSC activation likely through the interaction between Fyn and FAK, which activated FAK and led to the phosphorylation of N-WASP^23,24^. Fyn deficient mice showed alleviative CCl_4_ induced liver fibrosis. Importantly, we tested the anti-fibrotic effects of a clinically proven safe Fyn inhibitor Saracatinib in vitro and in vivo. Our results showed that Saracatinib significantly blocked the activation of both HSC cell lines and primary HSC cells. The oral administration of Saracatinib significantly attenuated liver fibrosis in a CCl_4_ induced liver fibrosis murine model. Saracatinib inhibits Fyn and other SFK members.

Up to now, there are nine members of SFKs: Src, Yes, Fyn, Fgr, Lyn, Hck, Lck, Yrk, and Blk were reported. Fyn is mainly expressed in brain, fibroblasts, endothelial cells, keratinocytes, T and B cells^7^. Fyn was the only SFK activated when HSCs were activated by TGFβ. However, little was known about the function of Fyn in HSCs. Our previous work focused on the role of activated Src in the transition from lung fibroblasts to myofibroblasts. The Src kinase inhibitor Saracatinib blocked TGF-β-induced Src kinase activation and α-SMA expression in lung fibroblasts^8^. Saracatinib can also effectively reduce lung fibrosis induced by Bleomycin^8^. Targeting Src was also effective in preventing the development of renal fibrosis^12^. For the Fyn, its role in liver fibrosis was not well documented. Seo et al. showed that mice lack of Fyn were resistant to renal fibrosis induced by unilateral ureteral obstruction^13^. Whether Fyn was involved in the activation of HSCs and liver fibrosis was unknown. We showed that Fyn was activated in HSCs with TGF-β treatment, mouse liver fibrosis model, and human liver fibrosis patients. Knockdown of Fyn inhibited the activation, proliferation, and migration of HSCs, moreover, mice with Fyn deficiency developed much less fibrosis in chronic CCl_4_ model. All these data strongly supported that Fyn was also critical in the pathogenesis of liver fibrosis.

The mechanism that how Fyn signaling contributes to the activation of HSCs is still unknown and will be studied in our future work. We and others demonstrated that FAK is required for TGF-β-induced α-SMA expression and the myofibroblasts differentiation^25–27^. Activated FAK directly bind to N-WASP and led to the phosphorylation of tyrosine residue 256 (Y256) of N-WASP^22,28^. It was well established that N-WASP was critical in filopodia formation and cell spreading^29–31^. Our previous study also supported that the deletion of FAK abolished phosphorylation of N-WASP and subsequently reduced α-SMA expression induced by TGF-β^22^. Based on these studies, we tested whether activated Fyn promoted the activation of HSC through the interaction with FAK and N-WASP. We showed that the knockdown of Fyn significantly reduced the activation of FAK (Fig. 2b). In addition, the treatment of Fyn inhibitor Saracatinib disrupted the interaction between Fyn and FAK as well as the activation of N-WASP, which may explain the prominent anti-fibrotic effects of Saracatinib and the potential mechanism by which Fyn activates HSCs.

Saracatinib was a novel SFK inhibitor, which is not only a potent target for c-Src, but also to other src family members: Fyn, c-Yes, Lyn, Blk, Fgr, and Lck^32^. Recently, Saracatinib was used for effectively treating the experimental models of lung fibrosis^8^. However, whether Saracatinib has any effect on liver fibrosis is still unknown. Although some common signaling pathways were involved in the development of liver fibrosis and lung/renal fibrosis, the unique features for HSCs, the ECM producing cells in the liver, have to be tested to understand whether Saracatinib can effectively block the activation of HSCs. Here we confirmed that Saracatinib showed potent inhibitory effects on the activation of HSC cell line, primary HSCs, and CCl_4_-induced liver fibrosis model. We also proposed that the anti-fibrotic effects of Saracatinib are mediated by the inhibition of Fyn signaling and disruption of the interaction between Fyn and FAK.

In summary, our results demonstrate that Saracatinib can be used in treating human liver fibrosis patients by targeting Fyn in HSCs, as the safety and pharmacokinetics of Saracatinib have been well documented^32^.

## Supplementary information

Supplemental figure legends

Figure S1

Figure S2

Figure S3

Supplemental Table 1
