# Supplementary material for: Targeting Src family kinase member Fyn by Saracatinib attenuated liver fibrosis in vitro and in vivo
Source: Cell Death Dis. 2020 Feb 12;11(2):118. doi: 10.1038/s41419-020-2229-2 (PMC7016006; doi:10.1038/s41419-020-2229-2)
Supplement: Supplementary file 1 — Supplemental figure legends [file 41419_2020_2229_MOESM1_ESM.docx]

**Figure S1 RNA-seq analysis of each member of Src family (FYN, SRC, LYN, LCK, *et, al*.) in human cirrhotic liver tissues.** RNA-seq raw reads were mapped to hg19. Transcripts were assembled using TopHat and quantified through Cufflinks. Fragments per kilobase of transcript per million mapped reads (FPKM) representing transcription level. (Mean ± SEM, N=4)

**Figure S2 Saracatinib has no obvious toxicity *in vivo* in the dose range. a** HE staining images showed that there was no significant damage in the liver of the mice in the simple administration group (Saracatinib 40 mg/kg/day) compared with the control group, (N=3). **b** Compared with the control group, there was no significant difference in the levels of ALT, AST, type IV collagen and hyaluronic acid in the serum of the Saracatinib group (N=3). **c** The body weight changes of the mice was measured every 3 days during the experiment (N=8).

**Figure S3 Saracatinib doesn’t affect the apoptosis of HSC cells.** Cells were seeded into 6-well plates and stimulated by Saracatinib for 12h. Then the cells were harvested and then incubated with Annexin V-FITC and PI staining buffer at room temperature for 15 min. The cell apoptosis was employed by a BD FACS Calibur (BD Biosciences). Data analysis was performed by FlowJo software (Tree Star Inc.).
