# Supplementary figures and images for: Targeting Src family kinase member Fyn by Saracatinib attenuated liver fibrosis in vitro and in vivo
Source: Cell Death Dis. 2020 Feb 12;11(2):118. doi: 10.1038/s41419-020-2229-2 (PMC7016006; doi:10.1038/s41419-020-2229-2)

**Figure S1**

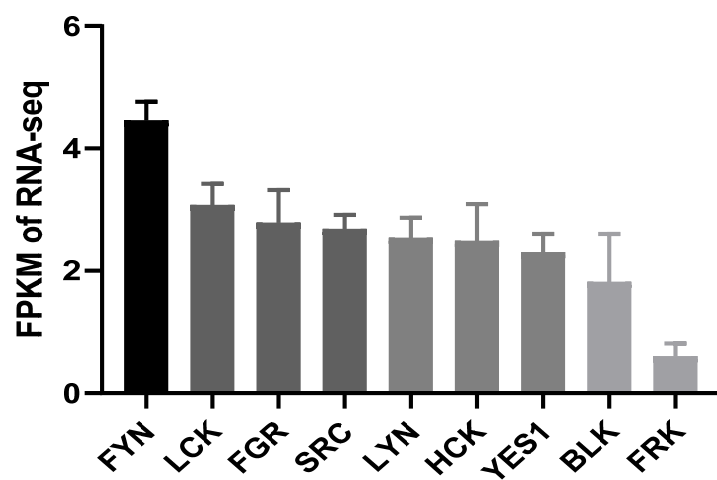

Supplement: Supplementary file 2 — Figure S1 [file 41419_2020_2229_MOESM2_ESM.pdf]

# Figure S2

**A**

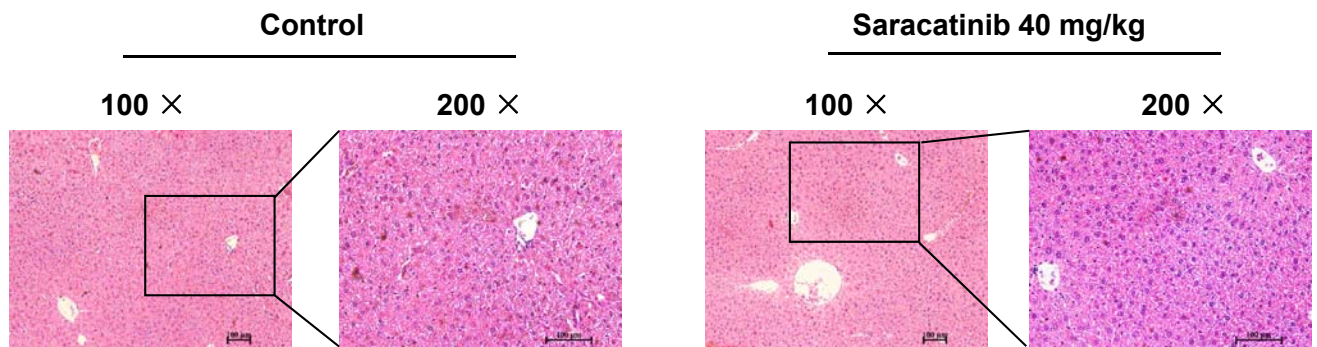

**B**

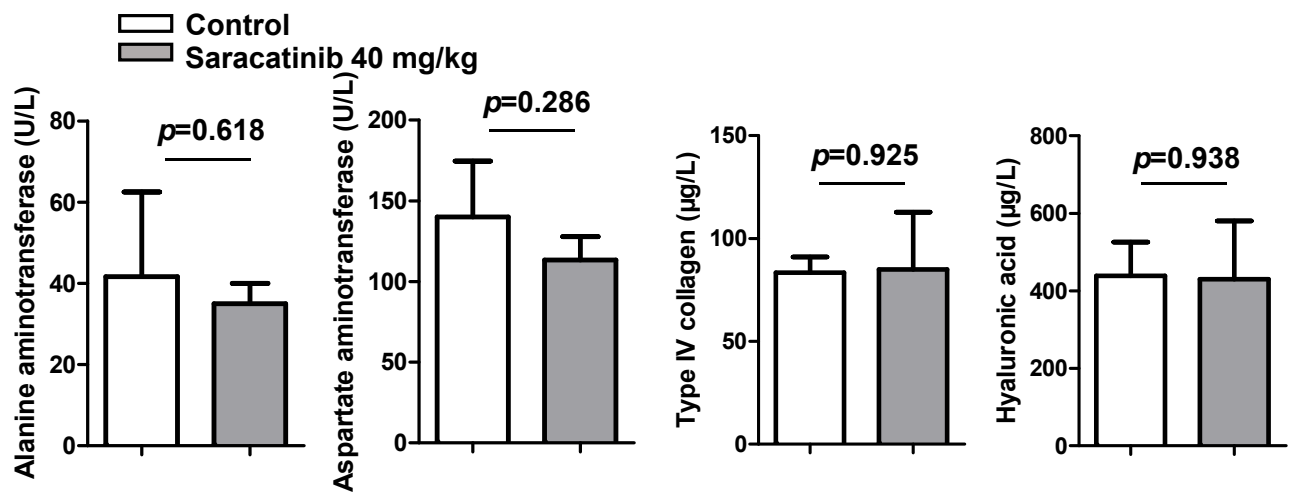

**C**

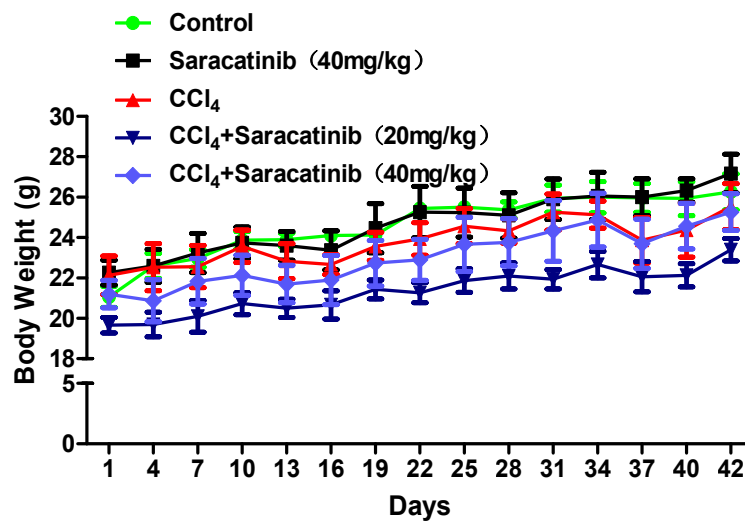

Supplement: Supplementary file 3 — Figure S2 [file 41419_2020_2229_MOESM3_ESM.pdf]

**Figure S3**

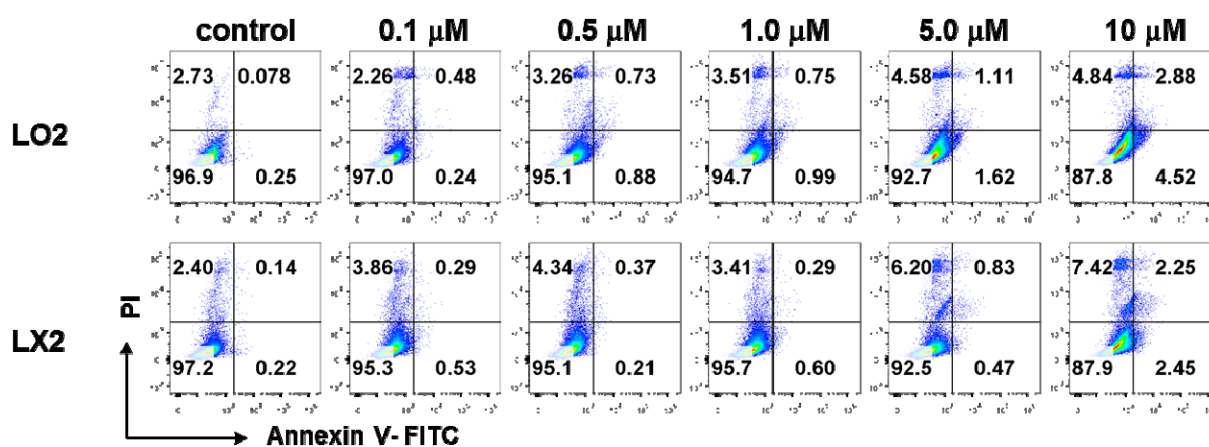

Supplement: Supplementary file 4 — Figure S3 [file 41419_2020_2229_MOESM4_ESM.pdf]
