# Supplementary material for: Targeting Src family kinase member Fyn by Saracatinib attenuated liver fibrosis in vitro and in vivo
Source: Cell Death Dis. 2020 Feb 12;11(2):118. doi: 10.1038/s41419-020-2229-2 (PMC7016006; doi:10.1038/s41419-020-2229-2)
Supplement: Supplementary file 5 — Supplemental Table 1 [file 41419_2020_2229_MOESM5_ESM.doc]

**Supplementary Table 1**: siRNA

| **Target** | **Sequence(5'-3')** |
| --- | --- |
| FYN-804-SENSE | GUGAACUCUUCGUCUCAUATT |
| FYN-804-ANTISENSE | UAUGAGACGAAGAGUUCACTT |
| FYN-1236-SENSE | GGUGGAUACUACAUUACCATT |
| FYN-1236-ANTISENSE | UGGUAAUGUAGUAUCCACCTT |
| FYN-1751-SENSE | GCGCAUGAAUUAUAUCCAUTT |
| FYN-1751-ANTISENSE | AUGGAUAUAAUUCAUGCGCTT |
| Negative control-SENSE | UUCUCCGAACGUGUCACGUTT |
| Negative control-ANTISENSE | ACGUGACACGUUCGGAGAATT |
